# Low Energy and Protein Intake in Brain Tumor Patients Despite Higher Adiposity: A Comparative Study with Gastrointestinal Cancer

**DOI:** 10.3390/nu18071051

**Published:** 2026-03-26

**Authors:** Innis Povazay, Leonie Burgard, Hans Joachim Herrmann, Markus Friedrich Neurath, Ilker Y. Eyüpoglu, Yurdagül Zopf

**Affiliations:** 1 Hector-Center for Nutrition, Exercise and Sports, Department of Medicine 1, Universitätsklinikum Erlangen, Friedrich-Alexander-Universität Erlangen-Nürnberg (FAU), 91054 Erlangen, Germany; 2 Department of Medicine 1, Universitätsklinikum Erlangen, Friedrich-Alexander-Universität Erlangen-Nürnberg (FAU), 91054 Erlangen, Germany; 3 Deutsches Zentrum Immuntherapie (DZI), Uniklinikum Erlangen, Friedrich-Alexander-Universität Erlangen-Nürnberg (FAU), 91054 Erlangen, Germany; 4 Department of Neurosurgery, Faculty of Medicine, University Hospital Carl Gustav Carus, Technische Universität Dresden, 01307 Dresden, Germany

**Keywords:** brain tumors, glioblastoma, cancer, oncology, protein intake, nutritional therapy, body composition

## Abstract

**Background/Objectives**: Nutritional therapy is an essential part of oncologic care, yet patients with brain tumors—especially those with glioblastoma—remain underserved by disease-specific dietary guidelines. This cross-sectional study compares energy and macronutrient intake, as well as body composition, between brain tumor patients, including a glioblastoma subgroup, and patients with gastrointestinal (GI) cancer. **Methods**: A total of 95 brain tumor patients and 109 GI cancer patients completed standardized three-day estimated food records and underwent bioelectrical impedance analysis and hand-grip strength measurements. Anthropometric parameters, as well as energy and macronutrient intake, were compared between groups. **Results:** Energy intake was lower in brain tumor patients compared with GI cancer patients (22.8 kcal/kg/day vs. 31.2 kcal/kg/day), as were protein (1.01 g/kg/day vs. 1.34 g/kg/day) and carbohydrates (182.8 g/day vs. 246.8 g/day; all *p* < 0.01). Despite the lower intake, brain tumor patients exhibited higher BMI scores, body fat percentages, and visceral fat levels (*p* < 0.05), while fat-free mass and skeletal muscle mass were comparable between groups. The phase angle was higher in brain tumor patients (*p* = 0.002), whereas the ECW/TBW ratio was lower (*p* = 0.003). In the glioblastoma subgroup, protein intake did not differ significantly compared with the GI cancer group. However, carbohydrate intake (190.9 g/day vs. 246.8 g/day; *p* = 0.01), as well as energy intake (25.7 kcal/kg/day vs. 31.2 kcal/kg/day; *p* = 0.05), remained significantly lower. **Conclusions**: Brain tumor patients were found to have energy intake levels below ESPEN recommendations for cancer patients (25–30 kcal/kg/day), and their protein intake was at the lower ESPEN threshold (1.0 g/kg/day), coupled with increased adipose tissue. The observed caloric deficit was accompanied by reduced carbohydrate intake, particularly in the glioblastoma subgroup. These findings highlight the need for individualized nutritional approaches in neuro-oncology. Until disease-specific recommendations are available, general dietary guidelines such as those by ESPEN offer a pragmatic interim orientation.

## 1. Introduction

The global incidence of cancer continues to rise, underscoring the growing importance of targeted supportive care measures, including nutritional therapy, as an integral part of comprehensive oncologic treatment [1,2]. Adequate nutritional support has been shown to positively influence functional status, quality of life, and overall prognosis in cancer patients [3,4]. This is particularly evident in gastrointestinal (GI) cancer, where maintenance of energy and protein intake is crucial to prevent or reduce cancer-related malnutrition and muscle wasting, which are strongly associated with increased morbidity and mortality [5,6,7].

Despite the established benefits of nutritional intervention in many tumor entities, including GI tumors, evidence-based guidelines remain scarce for people affected by brain tumors—especially glioblastoma, the most aggressive and common malignant brain tumor in adults [3,8,9]. Cancer patients are at high risk of developing tumor-associated cachexia characterized by involuntary weight loss, muscle wasting, and metabolic changes, which severely affect prognosis and quality of life. Whereas cachexia has been extensively studied in gastrointestinal cancers, much less is known about nutritional vulnerabilities in glioblastoma [2,10]. Due to neurological impairments, therapy-related side effects such as high-dose use of glucocorticoids and cognitive decline, glioblastoma patients, in particular, face unique nutritional challenges [11,12]. Nevertheless, unlike for patients with GI cancer, there is no consensus on optimal nutritional strategies in this population. As a result, many patients manage their illness with minimal dietary guidance, occasionally adopting individual approaches such as ketogenic diets, even though there is no or inconclusive evidence supporting their efficacy in glioblastoma and other brain tumors [13,14].

Given the impact of nutrition on patient outcomes and the existing gap in brain tumor-specific guidelines, further investigation on tumor-specific nutritional needs is urgently needed. Therefore, the aim of this cross-sectional study was to assess and analyze the energy and macronutrient intake and anthropometric parameters of brain tumor patients, with a particular focus on glioblastoma patients. By comparing these findings with data from patients with GI cancer—where more nutritional evidence exists—we aim to describe tumor-associated differences in intake and body composition. The goal is to assess potential nutritional inadequacies specific to glioblastoma patients, thereby exploring nutritional challenges in this population to inform future clinical nutrition research in neuro-oncology.

## 2. Materials and Methods

This comparative, cross-sectional study was conducted at the Hector-Center for Nutrition, Exercise, and Sports in the Department of Medicine 1 at the Uniklinikum Erlangen and the Department of Neurosurgery, Technische Universität Dresden, Faculty of Medicine, and at University Hospital Carl Gustav Carus, Dresden. Participants were recruited between July 2015 and April 2024. Ethics approval was obtained from the local ethics board (ethics approval numbers 155_13B and 79_19B for GI data and 3812 for brain tumor data). Reporting of this study adhered to the STROBE guidelines for cross-sectional studies [15,16]. The study included neurosurgically treated brain tumor patients across WHO CNS grades I–IV, as well as a comparative cohort of patients with various GI cancers, recruited during outpatient clinic visits. In addition to the primary comparison between the overall brain tumor cohort (WHO grades I–IV; *n* = 95) and the GI cancer cohort (*n* = 109), we performed a predefined subgroup analysis restricted to patients with glioblastoma (WHO grade IV; *n* = 30). This subgroup was compared with the same GI cancer cohort (*n* = 109). Brain tumor patients were included after confirmation of histopathological diagnosis and after surgery. They were assessed in a postoperative setting.

Inclusion criteria were: a confirmed diagnosis of brain tumors or GI cancer; age greater than 18 years; the ability to provide informed consent; and functional ability, as participants were required to independently complete dietary records and undergo functional assessments, including hand-grip strength testing and Karnofsky performance status assessment [17]. Exclusion criteria included bioelectrical impedance analysis (BIA) contraindications, as well as bedridden status. Members of the GI group were matched with brain tumor patients based on age and sex.

Dietary intake was recorded using a standardized three-day estimated food record, covering three consecutive days—two weekdays and one weekend day. Patients were instructed by trained dietitians on how to accurately document all food and beverage intake, including portion sizes, using the semi-quantitative “Freiburger Nutrition Protocol”, a prospective dietary assessment tool where intake is recorded directly after consumption, thereby minimizing recall bias. After completion, dietary records were reviewed by experienced nutrition staff, together with the patients, to ensure the completeness and plausibility of the reported intake. Goldberg cut-offs (as revised by Black) were applied, assuming a PAL of 1.4 [18]. The collected data were subsequently analyzed using the validated PRODI Expert Version 7.2 nutritional software (Nutri-Science GmbH, Freiburg, Germany) to quantify daily energy and macronutrient intake (carbohydrates, fats, and proteins) both in absolute terms and relative to body weight [19]. Since the fat mass differed significantly between the groups, energy and protein intake were additionally normalized to fat-free mass (FFM) and expressed as kcal/kg fat-free mass and g protein/kg fat-free mass, respectively.

Body composition and functional status were assessed through bioelectrical impedance analysis using a seca mBCA 515 BIA device (seca GmbH & Co. KG, Hamburg, Germany). This method provides data on body weight, body mass index (BMI), fat mass, fat-free mass, skeletal muscle mass, total body water (TBW), extracellular water (ECW), phase angle, and visceral fat [20]. We also report ECW/TBW as the extracellular water fraction. Measurements were conducted in the morning, in a fasted state, and prior to scheduled infusions by trained healthcare personnel following standardized protocols.

Hand-grip strength was measured as an indicator of functional muscle status. A calibrated grip-strength dynamometer was used under the supervision of trained healthcare personnel following standardized protocols. The highest value (in kg) from each hand was recorded, and the higher value was used for analysis [21].

We predefined age, sex and systemic glucocorticoid use as potential confounders due to their known influence on food intake, as well as body composition. Therefore, analyses were stratified by sex, and the GI cancer cohort was matched to the overall brain tumor cohort according to age. To further minimize bias, multiple procedures were implemented, such as staff training and timing standardization.

All statistical analyses were performed on a complete-case dataset using IBM SPSS Statistics (Version 30, IBM Corp., Armonk, NY, USA). No imputation was performed. Prior to hypothesis testing, data were examined for normality and homogeneity of variances using the Shapiro–Wilk test and Levene’s test, respectively. For between-group comparisons, independent-samples *t*-tests were used when the data met the assumptions of normality and equal variances. In cases where the assumption of normality was not met, non-parametric Mann–Whitney U-tests were applied as an alternative. These tests were used to compare outcomes between the groups. To account for multiple testing, Bonferroni correction was applied to between-group comparisons between the brain tumor and GI groups. All hypothesis tests were two-tailed, and a *p*-value <0.05 was considered statistically significant. Descriptive statistics are presented as means (95% CI). Additionally, Cohen’s d was calculated for key outcomes. Positive values indicate higher values for brain tumor patients compared with the GI group, and negative values indicate lower values [22].

## 3. Results

The cohort consisted of 95 brain tumor patients, of whom 65% were male and 35% were female (see Table 1). The comparison cohort consisted of 109 patients diagnosed with various types of GI cancers, of whom 59% were male and 41% were female, as shown in Table 1 and Table 2.

Functional status, assessed using the Karnofsky performance status, did not differ between groups (86 points in the brain tumor group vs. 84 points in the GI group).

Under-reporting was more frequent in the brain tumor cohort (26.3%) than in the GI group (12.0%). Over-reporting occurred in 7.0% of brain tumor patients and 7.0% of GI controls.

As shown in Table 2, the GI cohort consisted of a range of tumor entities across UICC stages I–IV.

The cohort consisted of patients across WHO CNS grades I–IV—Grade I: 12 (12.6%); Grade II: 24 (25.3%); Grade III: 29 (30.5%); Grade IV: 30 (31.6%)—with all WHO IV cases in our subgroup being glioblastomas (Table 3).

Systemic glucocorticoid use was recorded in 11 (11.6%) brain tumor patients, including 8 (26.7%) patients of the glioblastoma subgroup. Temozolomide treatment was documented in 52 (54.7%) brain tumor patients, with 28 (93.3%) in the glioblastoma subgroup.

### 3.1. Energy and Macronutrient Intake

The comparative analysis between brain tumor and GI cancer patients revealed several significant differences in energy and macronutrient intake and body composition, both overall and when stratified by sex (Table 4).

Compared with the GI cancer cohort, brain tumor patients had a significantly lower daily energy intake overall (*p* < 0.001; 1758 kcal/day vs. 2246 kcal/day; d = −0.73). This difference was found in both male (*p* = 0.002) and female patients (*p* = 0.02). Energy intake relative to body weight and fat-free mass was likewise significantly lower across sexes (all *p* ≤ 0.02).

Protein intake was also lower in brain tumor patients overall—including absolute (77.9 g/day vs. 97.5 g/day; d = −0.58), body weight-adjusted (1.01 g/kg/day vs. 1.34 g/kg/day; d = −0.66) and FFM adjusted (*p* < 0.004). In sex-stratified analyses, absolute protein intake was significantly lower in men with brain tumors than in men with GI cancer, whereas the difference in women did not remain significant after Bonferroni correction. Similarly, FFM-adjusted protein intake remained significantly lower only in men, with no significant differences in women, whereas body weight-adjusted protein intake was significantly lower in both men and women.

Carbohydrate intake was also significantly lower in brain tumor patients (*p* = 0.001), both in men (*p* = 0.002) and women (*p* = 0.01). Relative carbohydrate intake (as a proportion of total energy intake) was lower in brain tumor patients than in gastrointestinal cancer patients in the overall cohort; however, this difference was significant before Bonferroni correction (42.3% vs. 45.6%; *p* = 0.04) and did not remain significant after correction. With respect to absolute fat intake, a significant difference in absolute fat intake was observed between the brain tumor and GI groups (*p* = 0.01) but not in sex-stratified analyses. Although absolute macronutrient intakes differed significantly between groups, the relative macronutrient distribution (% of total energy intake) did not differ between groups after Bonferroni correction.

On average, brain tumor patients did not meet the ESPEN guidelines for energy and protein intake, as the mean intake was only 22.8 kcal/kg of body weight/day (recommended 25–30 kcal/kg/day), while protein intake was at the lower boundary of the recommended range (1.0 g/kg/day vs. 1.0–1.5 g/kg/day) [1].

Looking at the glioblastoma subgroup analysis (Table 5), energy intake was lower than in the GI cohort (*p* = 0.02; 1909 kcal/day vs. 2246 kcal/day). This difference persisted when normalized relative to body weight (25.7 kcal/kg/day vs. 31.2 kcal/kg/day; *p* = 0.05), as well as when adjusted for FFM. Protein intake did not differ significantly in either absolute or relative terms. Carbohydrate intake was significantly lower in the glioblastoma subgroup, both in absolute terms (*p* = 0.01; 190.9 g/day vs. 246.8 g/day) and relative to the other macronutrients (*p* = 0.03).

### 3.2. Body Composition and Hand-Grip Strength

Despite lower energy and protein intake, brain tumor patients showed a BIA phenotype indicative of higher adiposity compared with GI cancer patients (Table 6).

BMI (*p* = 0.001; 26.7 kg/m^2^ vs. 24.6 kg/m^2^; d = 0.46), fat mass (26.3 kg vs. 21.2 kg; d = 0.39), body fat percentage (32.0% vs. 28.3%), visceral fat (2.70 L vs. 1.90 L; d = 0.46) and phase angle (4.98° vs. 4.63°; d = 0.49) were significantly higher in the brain tumor group compared with the GI group, with differences remaining significant after Bonferroni correction. The ECW/TBW ratio was significantly lower in the brain tumor group.

In women, differences in BMI, fat mass, body fat percentage and phase angle remained significant after Bonferroni correction, while the difference in visceral fat was significant before but not after correction. ECW/TBW did not differ significantly between the brain tumor and GI groups in women.

In men, differences in fat mass, body fat percentage and ECW/TBW remained significant after correction; differences in several other values (e.g., visceral fat and phase angle) were significant before but not after Bonferroni correction.

No significant differences in hand-grip strength were observed between the brain tumor and GI groups.

In the glioblastoma subgroup, no statistically significant differences in body composition parameters were observed compared with the GI cohort (Table 7).

**Figure 1:**
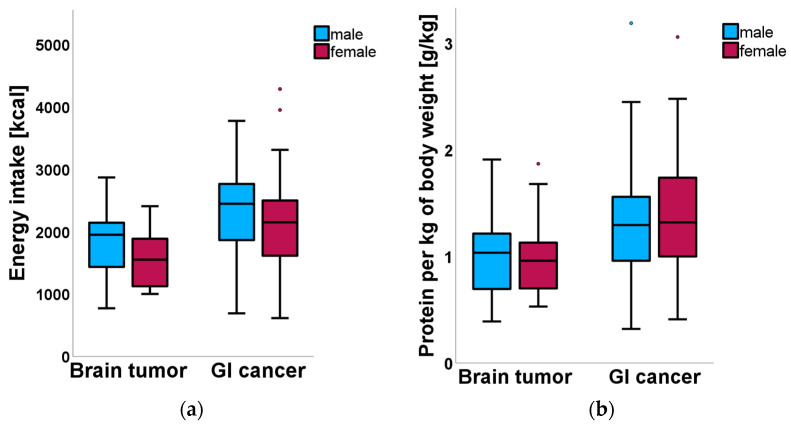
Comparison of dietary intake between patients with brain tumors and gastrointestinal (GI) cancer. Low Energy and Protein Intake in Brain Tumor Patients Despite Higher Adiposity: A Comparative Study with Gastrointestinal Cancer (July 2015 to April 2024). (**a**) Daily energy intake (kcal/day). (**b**) Daily protein intake per kg of body weight (g/kg/day). Data are presented as boxplots, with the center line indicating the median, the box representing the interquartile range, and the whiskers showing the range excluding outliers. Outliers are displayed as individual points.

**Figure 2:**
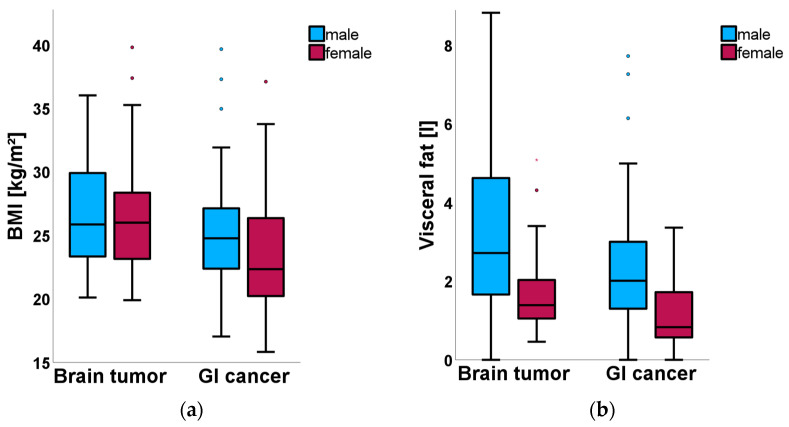
Comparison of body composition parameters between patients with brain tumors and gastrointestinal (GI) cancer. Low Energy and Protein Intake in Brain Tumor Patients Despite Higher Adiposity: A Comparative Study with Gastrointestinal Cancer (July 2015 to April 2024). (**a**) BMI (kg/m^2^). (**b**) Visceral fat (L). Data are presented as boxplots, with the center line indicating the median, the box representing the interquartile range, and the whiskers showing the range excluding outliers. Outliers are displayed as individual points and asterisks indicate extreme outliers.

To better visualize the results, Figure 1 summarizes energy intake and protein intake per kg of body weight by group and sex, whereas Figure 2 depicts body composition parameters such as BMI and visceral fat.

As illustrated in Figure 1a,b, brain tumor patients consumed significantly fewer calories than GI cancer patients, both in absolute and weight-adjusted terms. At the same time, they exhibited significantly higher BMI and visceral fat values (Figure 2a,b).

These findings show a pattern of hypocaloric intake, with protein intake at the lower boundary of ESPEN recommendations in the brain tumor group relative to GI patients, paired with greater adiposity but comparable lean mass.

**Figure 3:**
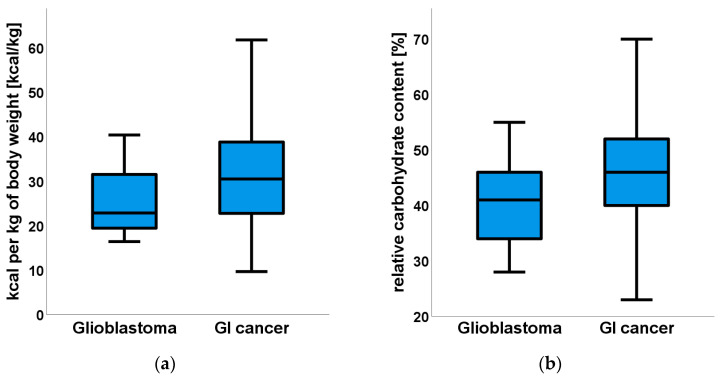
Comparison of dietary intake between patients with glioblastoma and gastrointestinal (GI) cancer. Low Energy and Protein Intake in Brain Tumor Patients Despite Higher Adiposity: A Comparative Study with Gastrointestinal Cancer (July 2015 to April 2024). (**a**) Daily energy intake per kg of body weight (kcal/kg/day). (**b**) Carbohydrate content relative to other macronutrients (%). Data are presented as boxplots, with the center line indicating the median, the box representing the interquartile range, and the whiskers showing the range excluding outliers.

Figure 3 shows that patients with glioblastoma had a lower energy intake per kilogram of body weight (kcal/kg/day) and a lower relative contribution of carbohydrates to total energy intake (%) compared with the GI cancer cohort.

## 4. Discussion

Our findings demonstrate a distinct nutritional and body composition profile in brain tumor patients compared with GI cancer patients. Although energy intake was significantly lower in the brain tumor cohort, these patients exhibited higher BMI and adiposity values while maintaining comparable lean mass. In particular, the observed hypocaloric intake and comparatively lower protein intake in the brain tumor cohort compared with GI cancer patients contrast with oncology nutrition guidelines, which emphasize adequate energy and protein intake to support muscle mass and functional status [1,4]. The macronutrient distribution in this cohort—characterized by relatively lower carbohydrate intake—may reflect patterns that partially overlap with carbohydrate-restricted dietary approaches. Across the spectrum of neurological diseases, dietary interventions—in particular, those involving carbohydrate restriction and the induction of a ketogenic state—have a long-standing clinical history [23]. In pediatric drug-resistant epilepsy, ketogenic dietary therapies are a well-established treatment modality supported by robust evidence [24].

In the context of brain tumors, carbohydrate-restricted or ketogenic approaches are increasingly discussed in clinical and patient-centered settings. This interest may partly stem from the established role of ketogenic dietary therapies in pediatric drug-resistant epilepsy, which has contributed to a broader association between carbohydrate restriction and neurological diseases. However, brain tumors differ fundamentally from epilepsy in their pathophysiology [13,23].

In clinical practice, patients may encounter dietary advice extrapolated from neurological or metabolic disease models (such as the Warburg effect), with an expectation of potential additive or supportive effects. Nevertheless, current evidence does not support ketogenic diets as a therapeutic intervention for brain tumor patients, including glioblastoma, and efficacy data remain insufficient to justify routine clinical recommendations [23]. A high-fat diet, however, may pose risks in oncology populations. Increased fat intake without adequate protein or total energy may promote adiposity without preserving lean mass, which could adversely affect prognosis [25,26]. The reduced total energy intake observed in the brain tumor cohort may not necessarily reflect tumor-specific metabolic adaptations but could also represent a broader pattern of dietary behavior shaped by commonly communicated recommendations within neurological care settings. Notably, the caloric deficit in our cohort appeared to be largely driven by lower carbohydrate intake, both in the overall brain tumor cohort and in the glioblastoma subgroup (Table 4 and Table 5).

These findings raise the possibility that reduced carbohydrate intake may represent a broader behavioral pattern across brain tumor patients spanning WHO grades I–IV, rather than being a phenomenon exclusive to glioblastoma. This shows that patients’ dietary habits often deviate from evidence-based oncologic nutrition and highlights the need to consider unintended consequences, such as insufficient energy intake [1].

The differences observed between brain tumor and GI patients may reflect differences in disease-related metabolic demands imposed by the two cancer types. GI cancer patients frequently experience severe malabsorption, gastrointestinal symptoms, and increased inflammatory burden, which lead to progressive cachexia and muscle loss [5,6,7]. In contrast, brain tumor patients, despite consuming fewer calories, maintain FFM and skeletal muscle mass at levels comparable to those of the GI group (Table 6), suggesting a lower degree of systemic inflammation. This interpretation is supported by higher phase-angle values in brain tumor patients (5.0° vs. 4.6°, *p* = 0.002), suggesting relatively better cell membrane integrity and nutritional status [11,20,27].

Nonetheless, increased adiposity—especially visceral fat—might be an overlooked risk factor for brain tumors. In breast cancer and other solid tumors, excess body fat and inflammation-related activity have been linked to worse treatment response and poorer survival [28,29,30]. Although such data are lacking for brain tumors such as glioblastoma, the higher levels of visceral fat in our brain tumor cohort (2.7 L vs. 1.9 L, *p* = 0.01) demand additional examination. The metabolic activity of visceral fat could potentially influence the tumor microenvironment and systemic therapy tolerance, as seen in other cancers [30,31].

Another notable observation concerns protein consumption. Although skeletal muscle mass did not differ substantially between groups, the reduced protein intake in brain tumor patients (1.01 g/kg/day vs. 1.34 g/kg/day, *p* = 0.003) may pose long-term risk for muscle catabolism and functional decline. Current European Society for Clinical Nutrition and Metabolism (ESPEN) guidelines recommend 1.0 g/kg/day to 1.5 g/kg/day protein intake for cancer patients to maintain muscle mass, particularly during treatment [1,3,32]. Mean protein intake was located at the lower boundary of ESPEN recommendations. Although formal thresholds were reached, intake at the minimal recommended level may not be sufficient for all patients, particularly during periods of metabolic stress.

Due to the cross-sectional design of this study, no causal conclusions can be drawn regarding whether the observed differences in energy and protein intake are a cause or a consequence of brain tumor-related disease burden. Reduced intake may reflect neurological deficits, treatment-related side effects, fatigue, or cognitive impairment rather than a primary disease-specific metabolic adaptation. Therefore, the findings should be interpreted as descriptive associations only. In addition, the gastrointestinal tumor entities included in our study are metabolically heterogeneous and may vary with regard to cachexia risk, disease stage at diagnosis, and nutritional burden, which should be taken into account when interpreting between-group comparisons. Despite their preserved lean mass, brain tumor patients consumed, on average, only 22.8 kcal/kg/day, which is below the 25–30 kcal/kg/day recommended for cancer patients, indicating a persistent caloric deficiency that could impair treatment outcomes [1]. In the glioblastoma subgroup, energy intake was also lower than in the GI cohort. This difference was largely attributable to lower carbohydrate intake (absolute and relative).

## 5. Limitations

This study has several limitations. First, under-reporting was frequent and particularly pronounced in the brain tumor cohort (26.3%) relative to the GI group (12.0%), which may have biased absolute intake estimates towards underestimation. However, the consistency of between-group differences across weight- and fat-free mass-adjusted analyses suggests that reporting bias alone is unlikely to fully explain the observed pattern. This may also partly reflect sarcopenic obesity, where reduced fat-free mass, alongside higher adiposity, can lead to overestimation of BMR by weight-based equations, as well as true energy undercoverage in brain tumor patients (rather than misreporting) due to treatment- and disease-related adverse effects (e.g., nausea, anorexia, fatigue, and taste changes) that can reduce intake despite a higher BMI [18]. Second, the GI cohort included diverse cancer entities and stages; limited sample sizes within subgroups prevented robust stratification, which may affect the interpretation of our findings. Dietary assessment was limited to total macronutrient intake. Carbohydrate quality (e.g., high vs. low molecular complexity) and protein composition, including branched-chain amino acids (BCAAs), were not assessed. Due to the cross-sectional nature of the study, causal relationships between dietary patterns and body composition cannot be confirmed. Even though BIA functions effectively in healthy people, cancer patients may have abnormal hydration levels, which can interfere with measurements [33,34]. A major potential confounder in the interpretation of body composition and hydration parameters is glucocorticoid therapy. High-dose corticosteroids are frequently used in glioblastoma patients to control cerebral edema and are known to induce hyperphagia, fat redistribution, fluid retention, and alterations in extracellular water compartments [35]. Consequently, the higher BMI, visceral fat, and phase angle observed in the glioblastoma group may, in part, reflect steroid-related metabolic and fluid shifts rather than superior nutritional status [36]. Importantly, conventional BIA-derived estimates of fat-free mass and related compartment measures rely on the assumption of a relatively fixed hydration of fat-free mass. Therefore, deviations from normal hydration (e.g., steroid-associated fluid retention) can bias the obtained body composition and hydration parameters [37].

Although our brain tumor cohort (*n* = 95) compares favorably to similar neuro-oncology studies, glioblastoma remains a rare tumor entity (*n* = 30). The subgroup analyses—especially when stratified by sex—are based on relatively small numbers, which reduces statistical power. Nonetheless, this study provides valuable insights into the nutritional status of patients with glioblastoma, a population for whom such data remain scarce. By highlighting distinct differences in energy and macronutrient intake and body composition profiles, the study generates important hypotheses regarding the metabolic characteristics of glioblastoma and underscores the need for individualized nutritional strategies. Future longitudinal and interventional studies should build upon these findings to explore causality and assess the clinical impact of targeted dietary interventions in neuro-oncology [38].

## 6. Conclusions

This cross-sectional study shows that patients with brain tumors consume significantly less energy and lower amounts of protein compared with patients with gastrointestinal tumors. Mean energy intake was below ESPEN recommendations, while protein intake was at the lower boundary of the recommended range [1]. Despite this hypocaloric and borderline protein-deficient diet, brain tumor patients had a higher BMI, greater total and visceral fat mass, and comparable fat-free mass. This suggests a specific body composition profile that differs from the typically cachectic phenotype seen in gastrointestinal tumors. In the glioblastoma subgroup, energy intake remained significantly lower than in the GI cohort, while differences in protein intake were not statistically significant. This suggests that reduced total energy intake—largely mirrored by lower carbohydrate intake—may represent a key dietary vulnerability in glioblastoma.

These findings underscore the need for systematic nutritional assessment in neuro-oncology. As part of routine care, brain tumor patients—especially those with glioblastoma—should undergo regular nutritional screening, including body composition analysis, and receive individual nutritional therapy counseling. Particular emphasis should be placed on achieving the ESPEN energy targets (25–30 kcal/kg/day) and adequate protein intake (1.0–1.5 g/kg/day), which can help maintain muscle mass, strengthen immune responses and mitigate treatment-induced catabolism [1,39]. Until meaningful disease-specific evidence is available, pragmatic, anti-inflammatory dietary patterns such as the Mediterranean diet and adherence to general oncological dietary guidelines appear more appropriate than restrictive approaches such as ketogenic diets with a lack of convincing evidence in brain tumor patients [40].

Future longitudinal and intervention studies are needed to determine the causal relationships between energy and protein intake, obesity, and clinical outcomes in brain tumors, particularly glioblastoma. Special attention should be paid to gender-specific differences in body composition and fat distribution, as well as to the potential prognostic value of detailed body composition parameters beyond BMI. Sociocultural, hormonal, or behavioral aspects may also contribute. Answering these questions is essential for the development of evidence-based, disease-specific nutritional guidelines for brain tumor patients.

## Figures and Tables

**Table 1:** Sample parameters. Low Energy and Protein Intake in Brain Tumor Patients Despite Higher Adiposity: A Comparative Study with Gastrointestinal Cancer (July 2015 to April 2024).

	Brain Tumor Cohort (*n* = 95)	GI Group (*n* = 109)
Male, *n* (%)	62 (65%)	64 (59%)
Female, *n* (%)	33 (35%)	45 (41%)
Age (years), mean (95% CI)	49.9 (47.1–52.8)	53.9 (51.8–55.9)
Body height (m), mean (95% CI)	1.73 (1.71–1.75)	1.73 (1.71–1.75)
Body weight (kg), mean (95% CI)	80.6 (76.9–84.3)	74.1 (70.4–77.2)
Karnofsky performance status, mean (95% CI)	85.8 (83.4–88.2)	83.6 (80.8–86.4)
Under-reporting	26.3%	12.0%
Over-reporting	7.0%	7.0%

**Table 2:** GI group classified by cancer type and stratified by sex and stage. Low Energy and Protein Intake in Brain Tumor Patients Despite Higher Adiposity: A Comparative Study with Gastrointestinal Cancer (July 2015 to April 2024).

*n*	Sex		Stage	
	Male	Female	Early Stage	Advanced Stage
Esophageal cancer	8	1	4	5
Pancreatic cancer	18	11	6	23
Gastric cancer	12	7	3	16
Colon cancer	22	22	6	38
Gallbladder cancer	1	4	3	2
Liver cancer	3	0	0	3

Early stage was defined as UICC stage I–II and advanced stage as UICC stage III–IV.

**Table 3:** Brain tumor parameters. Low Energy and Protein Intake in Brain Tumor Patients Despite Higher Adiposity: A Comparative Study with Gastrointestinal Cancer (July 2015 to April 2024).

	*n* (%)
WHO grade	
Grade I	12 (12.6)
Grade II	24 (25.3)
Grade III	29 (30.5)
Grade IV	30 (31.6)
Temozolomide therapy	
Yes	52 (54.7)
No	43 (45.3)
Systemic glucocorticoids	
Yes	11 (11.6)
No	84 (88.4)

**Table 4:** Comparison of daily energy and macronutrient intake between the brain tumor and GI groups (total sample). Low Energy and Protein Intake in Brain Tumor Patients Despite Higher Adiposity: A Comparative Study with Gastrointestinal Cancer (July 2015 to April 2024).

		Brain Tumor (*n* = 95) Mean 95% CI	GI Group (*n* = 109) Mean 95% CI	MD	d
Energy intake (kcal)	Total	1758 (1618–1898) *	2246 (2065–2374)	−488	−0.73
	Male	1856 (1681–2032) *	2342 (2134–2547)	−485	−0.76
	Female	1561 (1339–1784) *	2114 (1822–2290)	−553	−0.80
Body weight-adjusted energy intake (kcal/kg)	Total	22.8 (20.8–24.8) *	31.2 (28.6–33.7)	−8.4	−0.81
	Male	22.8 (20.2–25.5) *	29.7 (26.5–33.0)	−6.9	−0.68
	Female	22.7 (19.2–26.1) *	33.1 (29.0–37.2)	−10.5	−0.96
Protein (g)	Total	77.9 (71.1–84.8) *	97.5 (88.5–104.5)	−19.6	−0.58
	Male	82.3 (73.3–91.2) *	104.4 (92.7–115.7)	−22.1	−0.63
	Female	69.3 (59.0–79.7)	88.3 (75.7–96.5)	−19.0	−0.63
Body weight-adjusted protein intake (g/kg)	Total	1.01 (0.91–1.11) *	1.34 (1.22–1.45)	−0.33	−0.66
	Male	1.01 (0.88–1.13) *	1.31 (1.15–1.46)	−0.30	−0.62
	Female	1.02 (0.83–1.21) *	1.38 (1.19–1.56)	−0.36	−0.71
Protein En (%)	Total	18.6 (17.3–20.0)	17.8 (17.0–18.6)	0.8	0.20
	Male	18.6 (16.9–20.0)	18.1 (17.0–19.1)	0.5	0.12
	Female	18.6 (16.2–21.1)	17.3 (16.0–18.7)	1.3	0.30
Fat (g)	Total	71.5 (64.5–78.4) *	86.4 (77.8–93.6)	−14.9	−0.45
	Male	75.5 (65.9–85.1)	91.5 (81.2–101.3)	−16.0	−0.49
	Female	63.5 (55.6–71.5)	79.7 (65.4–91.0)	−16.2	−0.48
Fat En (%)	Total	37.7 (35.7–39.6)	35.2 (33.6–36.9)	2.4	0.33
	Male	37.3 (34.8–39.7)	35.9 (33.7–37.9)	1.4	0.19
	Female	38.5 (35.0–42.0)	34.4 (31.8–37.3)	4.1	0.52
Carbohydrates (g)	Total	182.8 (164.2–201.5) *	246.8 (224.7–261.3)	−64.0	−0.77
	Male	191.7 (169.6–213.9) *	251.8 (226.2–276.5)	−60.1	−0.76
	Female	165.0 (129.1–201.0) *	240.1 (214.5–258.9)	−75.0	−0.84
Carbohydrate (En%)	Total	42.3 (39.8–44.7)	45.6 (43.5–47.5)	−3.3	−0.36
	Male	42.3 (39.5–45.1)	44.6 (42.0–47.2)	−2.3	−0.26
	Female	42.2 (37.0–47.5)	47.0 (43.6–50.0)	−4.8	−0.49
FFM-adjusted energy intake (kcal/kg)	Total	32.8 (30.1–35.6) *	44.4 (40.9–47.9)	−11.6	−0.83
	Male	30.6 (27.5–33.8) *	39.8 (35.7–44.0)	−9.2	−0.76
	Female	37.2 (32.2–42.3) *	50.4 (44.7–56.1)	−13.2	−0.88
FFM-adjusted protein intake (g/kg)	Total	1.47 (1.32–1.62) *	1.90 (1.74–2.05)	−0.43	−0.65
	Male	1.36 (1.20–1.52) *	1.76 (1.56–1.96)	−0.40	−0.66
	Female	1.68 (1.37–1.99)	2.08 (1.82–2.33)	−0.40	−0.55

MD indicates the mean difference (brain tumor–GI group), and d = Cohen’s d is the standardized effect size. All intake variables are expressed per day. * Significant difference (*p* < 0.05 after Bonferroni correction) between brain tumor and GI groups.

**Table 5:** Comparison of daily energy and macronutrient intake between the glioblastoma and GI groups (total sample). Low Energy and Protein Intake in Brain Tumor Patients Despite Higher Adiposity: A Comparative Study with Gastrointestinal Cancer (July 2015 to April 2024).

	Glioblastoma (*n* = 30) Mean 95% CI	GI Group (*n* = 109) Mean 95% CI	MD	d
Energy intake (kcal)	1909 (1675–2143) *	2246 (2065–2374)	−337	−0.47
Body weight-adjusted energy intake (kcal/kg)	25.7 (21.6–29.7) *	31.2 (28.6–33.7)	−5.5	−0.48
Protein (g)	88.0 (75.7–100.2)	97.5 (88.5–104.5)	−9.5	−0.26
Body weight-adjusted protein intake (g/kg)	1.17 (0.99–1.35)	1.34 (1.22–1.45)	−0.17	−0.32
Protein En (%)	19.1 (17.0–21.1)	17.8 (17.0–18.6)	1.3	0.34
Fat (g)	78.2 (67.2–89.3)	86.4 (77.8–93.6)	−8.2	−0.23
Fat En (%)	38.4 (35.2–41.7)	35.2 (33.6–36.9)	3.2	0.43
Carbohydrates (g)	190.9 (158.4–223.3) *	246.8 (224.7–261.3)	−55.9	−0.64
Carbohydrate En (%)	40.4 (36.6–44.2) *	45.6 (43.5–47.5)	−5.2	−0.58
FFM-adjusted energy intake (kcal/kg)	35.3 (30.4–40.2) *	44.4 (40.9–47.9)	−9.1	−0.60
FFM-adjusted protein intake (g/kg)	1.62 (1.38–1.85)	1.90 (1.74 –2.05)	−0.28	−0.40

MD indicates the mean difference (glioblastoma–GI group), and Cohen’s d is the standardized effect size. All intake variables are expressed per day. * Significant difference (*p* < 0.05) between glioblastoma and GI groups.

**Table 6:** Comparison of body composition parameters between the brain tumor and GI groups (total sample). Low Energy and Protein Intake in Brain Tumor Patients Despite Higher Adiposity: A Comparative Study with Gastrointestinal Cancer (July 2015 to April 2024).

		Brain Tumor (*n* = 95) Mean 95% CI	GI Group (*n* = 109) Mean 95% CI	MD	d
BMI (kg/m^2^)	Total	26.7 (25.7–27.6) *	24.6 (23.6–25.3)	2.1	0.46
	Male	26.8 (25.7–28.0)	25.2 (24.3–26.4)	1.6	0.36
	Female	26.4 (24.6–28.2) *	23.6 (22.0–24.6)	2.8	0.58
Fat mass (kg)	Total	26.3 (23.9–28.7) *	21.2 (19.4–23.1)	5.1	0.39
	Male	25.0 (22.0–28.0) *	20.0 (17.7–22.4)	4.9	0.47
	Female	28.8 (25.0–32.6)*	22.9 (19.9–25.9)	5.9	0.58
Body fat percentage (%)	Total	32.0 (30.0–34.0) *	28.3 (26.6–30.1)	3.6	0.48
	Male	27.9 (25.7–30.0) *	23.8 (22.0–25.6)	4.0	0.51
	Female	39.7 (37.3–42.2) *	34.6 (32.3–37.0)	5.1	0.69
Fat-free mass (kg)	Total	54.3 (52.0–56.7)	52.6 (50.2–55.0)	1.7	0.14
	Male	61.0 (59.0–62.9)	60.9 (58.8–63.0)	0.0	0.01
	Female	41.9 (39.4–44.4)	41.1 (39.2–43.0)	0.8	0.12
Fat-free mass (index)	Total	17.9 (17.4–18.4)	17.3 (16.8–17.9)	0.5	0.21
	Male	19.1 (18.6–19.5)	19.0 (18.5–19.6)	0.0	0.02
	Female	15.7 (15.0–16.4)	15.0 (14.5–15.4)	0.7	0.41
Skeletal muscle mass (kg)	Total	25.8 (24.4–27.2)	23.9 (22.5–25.2)	1.9	0.27
	Male	29.4 (28.2–30.6)	28.4 (27.1–29.7)	1.1	0.22
	Female	18.7 (17.2–20.2)	17.5 (16.3–18.7)	1.2	0.30
Total body water (L)	Total	40.1 (38.4–41.9)	38.9 (37.1–40.5)	1.2	0.14
	Male	44.9 (43.4–46.4)	44.6 (43.0–46.2)	0.3	0.04
	Female	31.3 (29.4–33.2)	30.7 (29.3–32.2)	0.5	0.12
Extracellular water (L)	Total	17.6 (17.0–18.3)	17.6 (16.8–18.2)	0.1	0.02
	Male	19.3 (18.7–19.8)	19.7 (18.9–20.3)	−0.4	−0.16
	Female	14.6 (13.8–15.4)	14.6 (13.9–15.2)	−0.1	0.00
Phase angle (°)	Total	4.99 (4.83–5.13) *	4.63 (4.49–4.77)	0.4	0.49
	Male	5.20 (5.02–5.38)	4.93 (4.78–5.10)	0.3	0.40
	Female	4.58 (4.37–4.79) *	4.20 (4.02–4.38)	0.4	0.65
Visceral fat (L)	Total	2.70 (2.31–3.10) *	1.90 (1.60–2.21)	0.8	0.46
	Male	3.22 (2.69–3.75)	2.38 (1.93–2.83)	0.8	0.45
	Female	1.72 (1.33–2.11)	1.28 (0.96–1.60)	0.4	0.42
Maximum hand-grip strength (kg)	Total	37.8 (35.3–40.4)	40.7 (36.9–44.5)	−2.8	−0.23
	Male	43.5 (40.7–46.2)	46.8 (43.0–50.5)	−3.3	−0.32
	Female	27.3 (24.8–29.9)	27.4 (23.5–31.2)	−0.0	0.00
ECW/TBW	Total	0.444 (0.438–0.449) *	0.455 (0.450–0.461)	−0.01	−0.43
	Male	0.431 (0.426–0.436) *	0.441 (0.437–0.446)	−0.01	−0.55
	Female	0.467 (0.459–0.475)	0.476 (0.467–0.485)	−0.01	−0.34

MD indicates the mean difference (brain tumor–GI group), and Cohen’s d is the standardized effect size. * Significant difference (*p* < 0.05 after Bonferroni correction) between brain tumor and GI groups.

**Table 7:** Comparison of body composition parameters between the glioblastoma and GI groups (total sample). Low Energy and Protein Intake in Brain Tumor Patients Despite Higher Adiposity: A Comparative Study with Gastrointestinal Cancer (July 2015 to April 2024).

	Glioblastoma (*n* = 30) Mean 95% CI	GI Group (*n* = 109) Mean 95% CI	MD	d
BMI (kg/m^2^)	25.3 (23.9–26.7)	24.6 (23.6–25.3)	0.7	0.16
Fat mass (kg)	23.9 (20.3–27.6)	21.2 (19.4–23.1)	2.7	0.28
Body fat percentage (%)	30.9 (27.0–34.8)	28.3 (26.6–30.1)	2.5	0.27
Fat-free mass (kg)	52.6 (48.6–56.7)	52.6 (50.2–55.0)	0.0	0.00
Fat-free mass (index)	17.3 (16.4–18.2)	17.3 (16.8–17.9)	−0.1	−0.02
Skeletal muscle mass (kg)	24.8 (22.5–27.1)	23.9 (22.5–25.2)	0.9	0.13
Total body water (L)	38.9 (35.9–41.8)	38.9 (37.1–40.5)	0.0	−0.01
Extracellular water (L)	17.5 (16.4–18.5)	17.6 (16.8–18.2)	−0.1	−0.03
Phase angle (°)	4.62 (4.29–4.94)	4.63 (4.49–4.77)	−0.01	−0.01
Visceral fat (L)	2.60 (1.87–3.32)	1.90 (1.60–2.21)	0.7	0.43
Maximum hand-grip strength (kg)	35.2 (30.0–40.5)	40.7 (36.9–44.5)	−5.4	−0.42
ECW/TBW	0.455 (0.443–0.468)	0.455 (0.450–0.461)	0.00	−0.01

MD indicates the mean difference (glioblastoma–GI group), and Cohen’s d is the standardized effect size.

## Data Availability

The data presented in this study are available upon request from the corresponding author due to privacy and ethical restrictions on patient information.

## Abbreviations

The following abbreviations are used in this manuscript:

BIA	Bioelectrical impedance analysis
BMI	Body mass index
FFM	Fat-free mass
FFMI	Fat-free mass index
TBW	Total body water
ECW	Extracellular water
GI	Gastrointestinal
MD	Mean difference
d	Cohen’s d
*p*	*p*-value
